# CD43 Promotes Cells Transformation by Preventing Merlin-Mediated Contact Inhibition of Growth

**DOI:** 10.1371/journal.pone.0080806

**Published:** 2013-11-18

**Authors:** Nohemi Camacho-Concha, Amiel Olivos-Ortiz, Alfredo Nuñez-Rivera, Adolfo Pedroza-Saavedra, Lourdes Gutierrez-Xicotencatl, Yvonne Rosenstein, Gustavo Pedraza-Alva

**Affiliations:** 1 Departamento de Medicina Molecular y Bioprocesos, Instituto de Biotecnología, Universidad Nacional Autónoma de México, Cuernavaca, Morelos, México; 2 Centro de Investigación Sobre Enfermedades Infecciosas, Instituto Nacional de Salud Pública, Cuernavaca, Morelos, México; Case Western Reserve University, United States of America

## Abstract

In normal tissues, strict control of tissue size is achieved by regulating cell numbers. The mechanism that controls total cell number is known as contact inhibition of growth and it depends on the NF2/Merlin pathway. Negative regulation of this pathway by deleterious mutations or by oncogenes results in cell transformation and tumor progression. Here we provide evidence that the CD43 sialomucin cooperates with oncogenic signals to promote cell transformation by abrogating the contact inhibition of growth through a molecular mechanism that involves AKT-dependent Merlin phosphorylation and degradation. Accordingly, inhibition of endogenous CD43 expression by RNA interference in lung, cervix and colon human cancer cells impaired tumor growth *in vivo*. These data underscore a previously unidentified role for CD43 in non-hematopoietic tumor progression.

## Introduction

Cell transformation leading to tumor formation is a multi-step process that results from the accumulation of mutations that favor cell survival, unlimited cell perpetuation, auto- sufficiency and general unresponsiveness to environmental signals [1]. A key element to tissue cell number control is the mechanism known as cell contact inhibition of growth, where cells proliferate until they occupy the available space and stop when contact with other cells or dense extracellular matrix is tight. Not surprisingly, numerous tumors carry mutations in components of the signal transduction pathway that controls contact inhibition of growth [2]. This pathway, known as the Hippo pathway, is initiated when the tumor suppressor Merlin, encoded by the Nf2 gene [3], switches from a closed conformation, imposed by p21-PAK kinase phosphorylation on Ser518, to an active open conformation resulting from PAK inhibition by cadherin-mediated adhesion [4]. Active Merlin leads to the activation of the Mst1/2 kinase, which phosphorylates and activates Lats1/2 kinase through a yet poorly defined mechanism, ultimately leading to YAP phosphorylation [5]. YAP phosphorylation on Ser127 residue leads to its interaction with 14.3.3 proteins and its retention in the cytosol [6]. The subsequent phosphorylation of Ser381 results in YAP ubiquitylation and degradation [7], preventing the expression of YAP target genes involved in cell proliferation and survival. Mitogenic signals provided by the epidermal growth factor receptor (EGFR) or integrins target Merlin [8], promoting cell proliferation. Consequently, negative regulation of this pathway is crucial for tumor development.

CD43 is a highly glycosylated type I transmembrane protein with a mucin-like structure expressed in all immune cells, but mature naïve B lymphocytes. Long considered to be exclusively expressed in cells from the hematopoietic and immune systems, recent reports underscore a role for CD43 in non-immune cells as its expression, both at the mRNA and protein level, has been documented in different human non-lymphoid tumor cells [9,10] as well as in rat kidney and brain epithelium [11]. Although CD43 functions in the immune response have been well described [12], the role of this sialomucin in tumor biology starts only to be elucidated. It has been suggested that, similar to Notch, in human colon tumor cells CD43 undergoes a proteolytic cleavage that results in the translocation of its intracellular domain to the nucleus where it interacts with β-catenin and TCF, promoting c-Myc and Cyclin D expression, leading to cell proliferation [13]. In addition, the interaction of CD43 with ICAM-1 or E-selectin at late stages of tumor development promotes metastasis [14]. Nonetheless, the mechanisms through which CD43 contributes to the transformation process and tumor development remain largely unknown.

Using loss and gain of function approaches, we show that CD43 cooperates with oncogenic signals to promote cell transformation by abrogating Merlin growth-suppressive functions through an AKT-dependent mechanism.

## Materials and Methods

### Mice

nu/nu mice were obtained from the Jackson laboratory. Animals were maintained in our animal facility in a ventilated rack with food and water *ad libitum*. Experiments were carried according to institutional guidelines and approved by The Bioethics Committee of the Instituto de Biotecnología, UNAM.

### Cell lines

The human tumor derived cell lines A549 (lung); CasKi (cervix) and DLD-1 (colon) were obtained from the ATTC and maintained in culture following ATCC instructions. NIH-3T3 fibroblasts expressing the human EGFR (NIH-3T3-hEGFR) [15,16] and transgenic mouse fibroblasts expressing the E6/E7 oncoproteins from the human papillomavirus type 16 (HPV16) [17] were cultured in DMEM supplemented with 10% fetal calf serum (FCS, Invitrogen), 2 mM L-glutamine (Sigma), 50 U/ml penicillin and 50 μg/ml streptomycin (Invitrogen).

### Antibodies

L10, an IgG1 mAb that recognizes CD43 has been described previously [18]. The anti-Myc, -EGFR, -Cyclin D, -Merlin, -ERK, -p-AKT, -actin and -GFP antibodies were from Santa Cruz Biotechnology. The anti-p-STAT, -p-YAP and -p-GSK3β antibodies were from Cell Signaling.

### CD43 expression

NIH-3T3-hEGFR or E6/E7 fibroblasts were transfected using Lipofectamine 2000 (Invitrogen) and 5 μg of the linear pFNeo expression vector, empty or containing the CD43 wild-type (Wt) cDNA or a previously reported [19] CD43 mutant lacking the intracellular domain (∆IC). Single clones were obtained by G418 (800 μg/ml) selection. All biological assays were performed with at least four independent clones expressing the Wt or mutated forms of CD43 or the empty vector (pFNeo).

### RNA interference

A synthetic oligonucleotide coding for an interfering RNA specific for the human CD43 mRNA (AA ATG GCC ACG CTT CTC CT) was cloned into the BglII-Sal1 sites of the pSuper/EGFP plasmid [20]. DraIII linearized DNA (5 μg) was transfected as above, clones were obtained by G418 selection (800 μg/ml). GFP positive single clones were isolated and CD43 expression levels were evaluated by immunoblot; at least four independent clones with normal (pSup) or low (RNAi) CD43 expression levels were used for all biological assays. To reduce Merlin protein levels in A549 clones with low CD43 expression (RNAi), cells were transfected with the siRNA smart pool for human Nf2 mRNA from Dharmacon using Lipofectamine; reduction of Merlin protein levels was confirmed by immunoblot.

### Wound healing assay

Cells were cultured in 35 mm diameter dishes until they reached confluence. The monolayer was rinsed with PBS and cultured for an additional 12 hrs with serum-free media. Then, complete media was added and the monolayer was wounded with a yellow pipette tip. Cells were cultured until control cells closed the wound (100% healing); the area not healed in the test plates was measured and reported relative to 100% healing. The PI3K inhibitor LY294002 was added to the cultures for the indicated time at 20 μM final concentration. 

### Soft agar assay

Cells (1x10^6^ or 1x10^5^) were seeded in triplicate plates and grown in soft agar as previously described [16] for 21 days. Colonies were counted under a light microscope. 

### Monolayer colony formation

After cells reached confluence, media was changed every other day and cells were cultured until foci were evident, at which point, cells were fixed with 3.7% formaldehyde in PBS, stained with Giemsa and counted [21].

### *In vivo* tumor formation

A549 cells (1x10^6^) CasKi (3x10^6^) or DLD-1 cells (1x10^6^) expressing the CD43 RNAi or containing the empty pSuper vector, and NIH-3T3 (3x10^6^), NIH-3T3-hEGFR (3x10^6^) or E6/E7 fibroblasts (3x10^6^) expressing the Wt, the mutated CD43 molecule or the empty pFNeo vector were injected subcutaneously to six weeks old female nu/nu mice. After one month, animals were sacrificed, the tumor was surgically excised and its weight was determined. 

### Cell proliferation

2x10^4^ cells were seeded in 24 well plates or 35 mm plates and cultured for the indicated times in supplemented medium, cells were harvested with trypsin, washed and counted. Where indicated, cells were allowed to reach confluence and new media was added, following which cells were then cultured for the indicated period of time in the presence or absence of the PI3K inhibitor LY294002 (20 μM).

### Immunoprecipitation and Immunoblotting

Cells were lysed in 100 μl of lysis buffer (20 mM Tris pH 7.4, 137 mM NaCl, 2 mM PPiNa, 2 mM EDTA, 1% Triton X-100, glycerol 10%, 0.5 mM DTT, 25 mM β-glycerophosphate, 200 mM Na_3_VO_4_, 1 mM PMSF, 5 μg/ml leupeptin, 5 μg/ml aprotinin, 5 μg/ml antipain) for 10 min at 4°C. Lysates were spun at 14,000xg for 15 min at 4°C and kept at -70°C until use. Immunoprecipitation and blotting were performed as previously described [22]. 

### Statistical analysis

Data shown are the mean ± SD; they were analyzed by ANOVA, considered significant at p < 0.05.

## Results

### CD43 signaling cooperates with oncogenic signals to promote cell transformation

**Figure 1:**
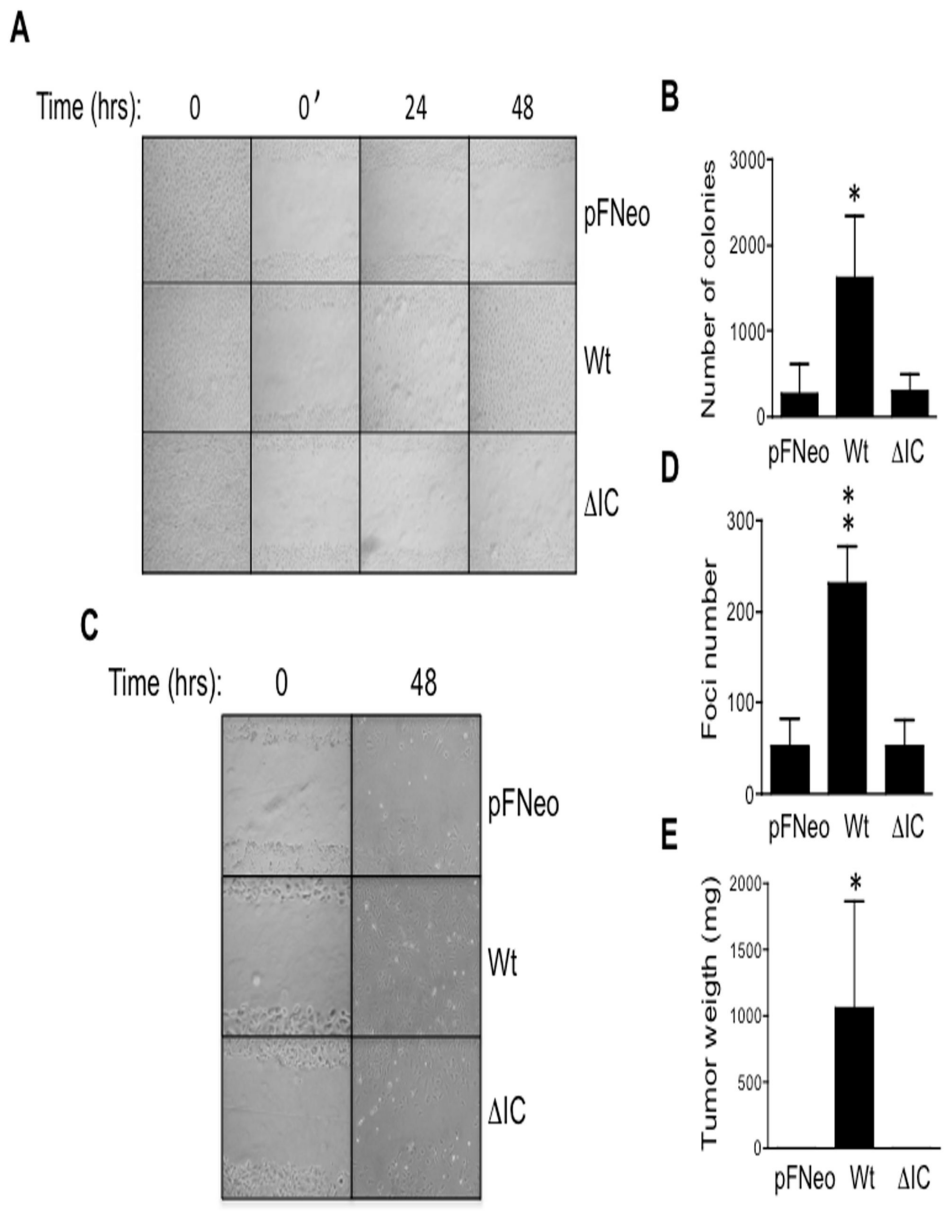
CD43 signaling cooperates with oncogenic signals to promote cell transformation. NIH-3T3-hEGFR (**A**) or E6/E7 transgenic mouse fibroblasts (**C**) carrying the pFNeo empty vector (pFNeo), expressing wild-type CD43 (Wt) or CD43 lacking the intracellular domain (ΔIC) were grown to confluence; the monolayer was then wounded (t=0) and healing was evaluated by light microscopy at the indicated time points. NIH-3T3-hEGFR fibroblasts were grown in soft agar as described in material and methods; after three weeks, colonies were counted (**B**). E6 transgenic mouse fibroblasts stably transfected with the indicated constructs were grown to confluence for three weeks; foci were stained with Giemsa and counted (**D**). 3X10^6^ E6/E7 fibroblasts stably transfected were injected subcutaneously into nu/nu mice; one month later, animals were sacrificed and tumor mass was weighed (**E**). Data shown are representative of at least four independent experiments performed with at least four independent pFNeo, Wt or ΔIC clones from each cell line. Graphs represent the average cell number ± SD of three independent experiments using at least 3 independent clones. *p < 0.05 vs pFNeo.

Although it is well documented that different non-lymphoid tumors express CD43 [10], the role for this mucin in cell transformation is not entirely elucidated. Exogenous CD43 expression in non-hematopoietic cells containing ARF and p53 mutations has been shown to result in cell proliferation [23]. In contrast, in cells expressing wild-type ARF and p53, CD43 expression leads to cells death [24]. This suggests that CD43 requires additional oncogenic signals to promote cell proliferation in non-hematopoietic cells. To test whether CD43 signals could favor cell transformation in conjunction with a given oncogenic signal, we expressed the human CD43 molecule in mouse NIH-3T3 fibroblasts expressing the human EGFR [16] or in fibroblasts derived from a transgenic mouse expressing the E6/E7 oncoproteins from HPV16 [17]; both cell lines express wild-type p53. Clones co-expressing CD43 and the EGFR showed an enhanced capability to close the wound in a wound healing assay (Figure 1A) and formed more (Figure 1B) and bigger colonies in soft agar assays than cells expressing the EGFR alone (Figure S1, upper panel). This was not the result of differences in EGFR expression levels, as EGFR expression was similar in CD43^-^ and CD43^+^ clones (Figure S2). Moreover, when the CD43 intracellular domain was missing (ΔIC), both the wound healing and anchorage-independent growth capacities were lost (Figure 1A and 1B). Similarly, cells co-expressing CD43 together with the oncogenic proteins E6/E7 from HPV16 closed wounds faster than cells carrying the empty vector (Figure 1C) and formed more foci when in confluence (Figure 1D and Figure S1, lower panel); likewise, this required the intracellular domain of CD43 (Figure 1C and 1D). 

To test whether CD43 expression promoted tumor formation *in vivo*, nude mice were injected subcutaneously with E6/E7 transgenic mouse fibroblasts expressing wild-type CD43 or CD43 lacking the intracellular domain (ΔIC), and the weight of the tumor mass was evaluated after one month. In agreement with data presented above, CD43 expression promoted tumor formation *in vivo* only when wild-type CD43 was expressed in E6/E7 fibroblasts. On the contrary, tumors did not develop when cells co-expressed the mutant CD43 molecule lacking its intracellular domain (ΔIC) with the E6/E7 oncoproteins or the E6/E7 oncoproteins alone (pFNeo, Figure 1E). As previously published [24], expression of CD43 alone in NIH-3T3 fibroblast (Figure S3A) did not promote cell transformation by itself, since similar to CD43^-^ cells (empty vector), the Wt CD43^+^ cells did not close the wound after 72 hrs (Figure S3B), time at which cells start to die. Although, wild-type CD43^+^ NIH-3T3 cells formed more colonies in soft agar than cells carrying the pFNeo empty vector or expressing the CD43 mutant lacking the intracellular domain (Figure S3C), those colonies were smaller than the ones formed when CD43 was expressed together with the EGFR (data not shown). Furthermore, no significant difference in the proliferative capacity of Wt CD43^+^ and CD43^-^ NIH-3T3 fibroblasts was observed (Figure S3D). Accordingly, the tumor size in nude mice injected with CD43^+^ NIH-3T3 fibroblasts was very similar to that of mice injected with CD43^-^ NIH-3T3 fibroblasts (Figure S3E). Together, these results suggest that CD43 signals cooperate with oncogenic signals to favor cell transformation and that the CD43 intracellular domain is required for this process.

### CD43 expression contributes to tumoral fitness of human derived tumor cells

**Figure 2:**
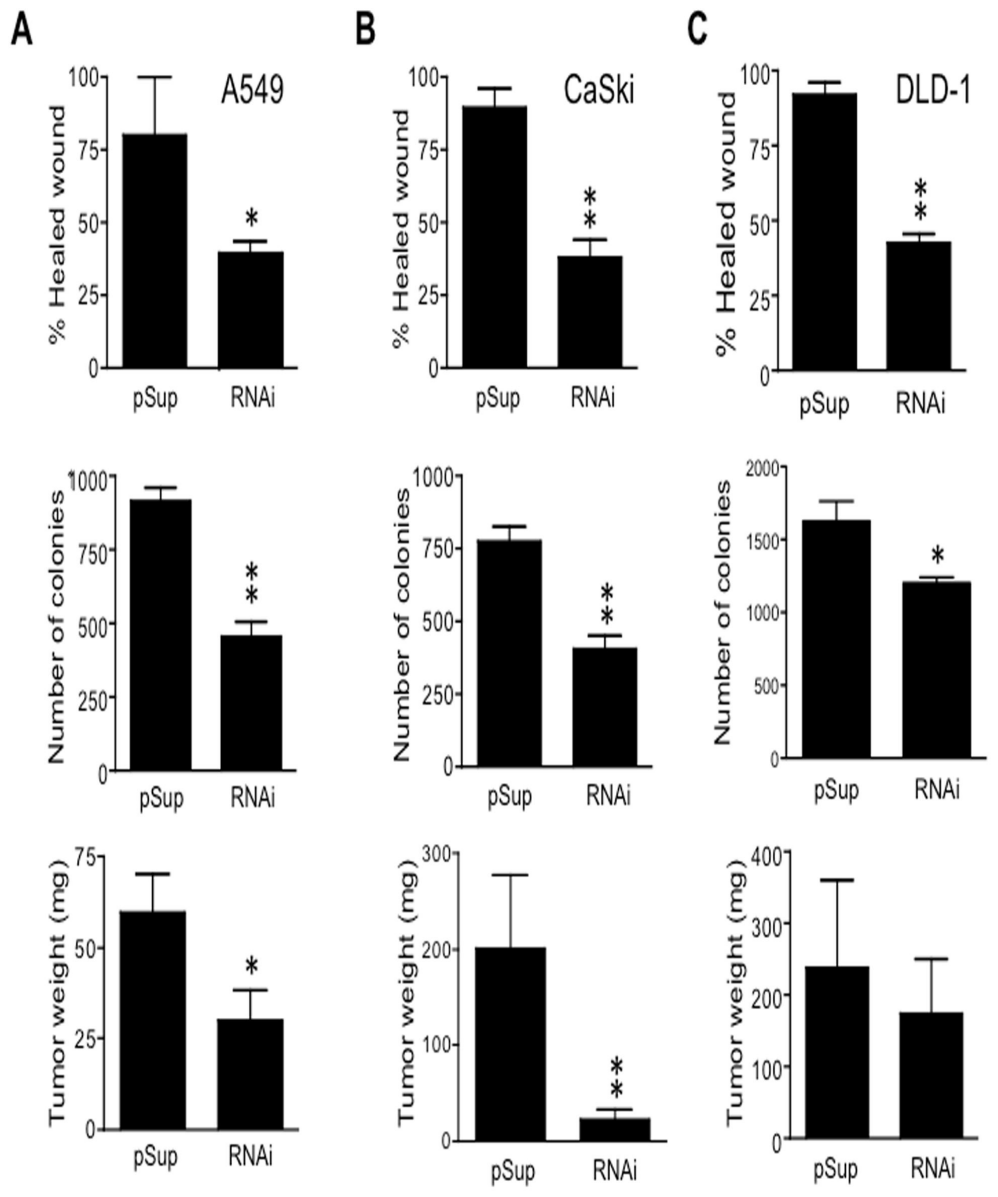
CD43 expression confers tumoral fitness to human tumor-derived cells. A549 lung (**A**), CasKi cervix (**B**) or DLD-1 colon (**C**) tumor cells containing the empty pSuper (pSup) vector or expressing the CD43 specific RNAi (RNAi) were cultured to confluence, the monolayer was then wounded and healing was evaluated (upper panel). Cells were also cultured in soft agar as indicated in material and methods, after three weeks colonies were counted (middle panel). Cells (1X10^6^ for A549, 3X10^6^ for CasKi or DLD-1) were injected subcutaneously into nu/nu mice; one month later, animals were sacrificed and tumor weight was evaluated (lower panel). Data represents the average ± SD of four independent experiments performed with four independent pSup or RNAi clones for each cell line. *p < 0.05, **p < 0.01 vs pSup.

CD43 is expressed in different human tumors [25] as well as in cell lines derived from lung carcinoma (A549), cervix (CasKi) and colon (DLD-1) [10]. To test whether expression of CD43 favored any tumor hallmark in these tumor cell lines, we reduced CD43 expression levels by RNA interference (Figure S4A and 4B). In accordance with the results obtained from the gain of function experiments shown above, clones with reduced CD43 expression levels derived from the A549 lung carcinoma cell line showed a 50% reduction in their wound healing capacity (Figure 2A, upper panel), and formed less and smaller colonies in soft agar than their counterparts with normal CD43 levels (Figure 2A, middle panel). In agreement with data showing that CD43 signals cooperate with the E6/E7 oncoproteins from HPV16 to favor cell transformation (Figure 1E), decreasing CD43 expression levels in the cervical tumor cells line CasKi resulted in approximately 50% reduction in the wound healing capacity (Figure 2B, upper panel) as well as in its anchorage-independent growth capacity (Figure 2B, middle panel), compared with clones expressing normal CD43 levels. As CD43 has been shown to favor cell proliferation of colon derived tumor cells, we assessed whether CD43 would provide an advantage to the tumor cell line DLD-1, a cell line derived from a stage 3 colorectal cancer. Similar to A549 and CasKi cells, reduction of CD43 expression levels in DLD-1 cells also resulted in decreased wound healing (Figure 2C upper panel) and substrate-independent growth capacities (Figure 2C, middle panel). Collectively, these results suggest that during carcinogenesis, expression of CD43 provides signals that promote cell transformation.

To validate the idea that CD43 expression favors the tumorigenic potential of human derived cancer cells, nude mice were injected subcutaneously with A549, CasKi or DLD-1 cells expressing normal or low CD43 levels (RNAi, CD43^low^) and the number of mice that developed a tumor as well as the tumor weight were evaluated. Independent of the cell line, reduction of CD43 expression levels did not prevent tumor formation, since all mice (n=8) inoculated with CD43^low^ cells developed tumors (Figure S4C). Nonetheless, impairing CD43 expression resulted in significantly smaller tumors, independently of the tissue origin (lung, < 50%; cervix, < 80%) as compared to tumors derived from cells expressing normal CD43 levels (Figure 2A and 2B, lower panel). However, reducing CD43 expression in the colon DLD-1 tumor cells only partially affected tumor size (<30%) (Figure 2C, lower panel). Altogether, these results show that CD43 expression potentiated the tumorigenic capacity of non-hematopoietic human tumor derived cell lines. 

### The CD43 intracellular domain is required to promote cell transformation

**Figure 3:**
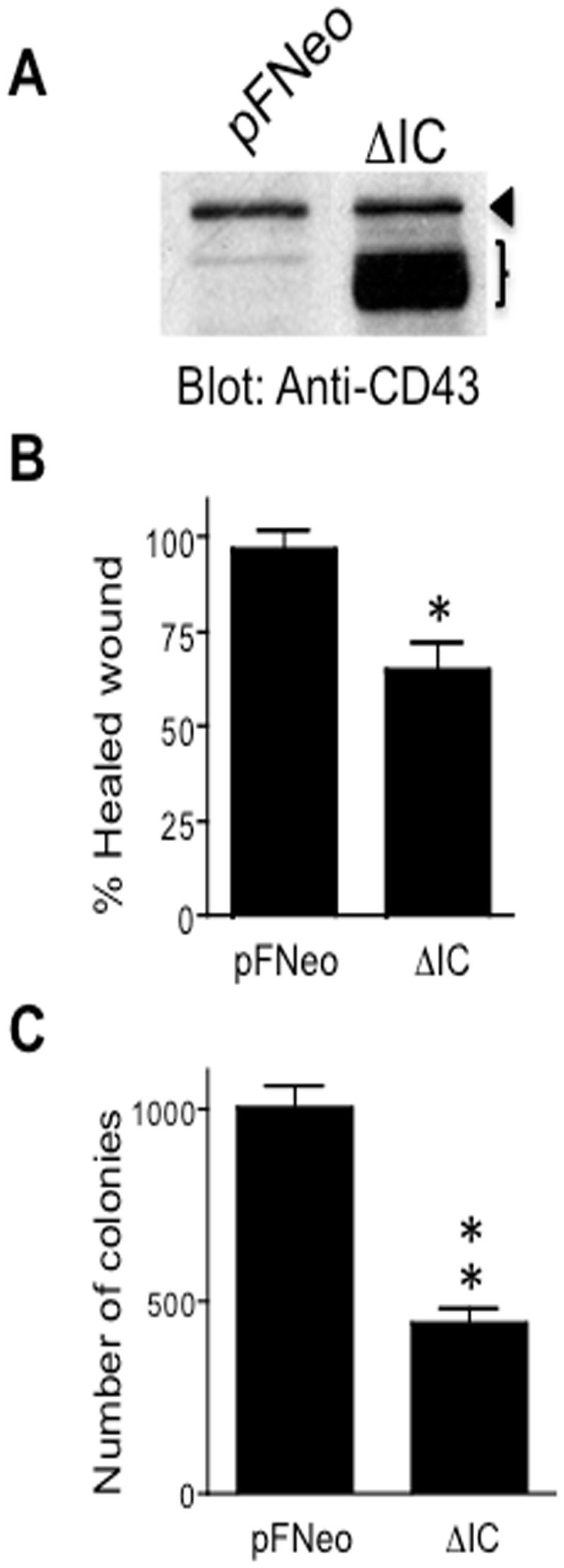
CD43 intracellular domain is required to promote cell transformation. Total cell extracts from A549 lung tumor cells stably containing the empty pFNeo vector (pFNeo) or expressing CD43 lacking the intracellular domain (ΔIC) were resolved on SDS-PAGE and transferred to nitrocellulose membranes. CD43 expression levels were determined by immunoblot using anti-CD43 specific antibodies (**A**). The arrowhead indicates the endogenous wild-type CD43 molecule; the bracket, the CD43 mutant lacking the intracellular domain. A549 lung tumor cells were grown to confluence, the monolayer was then wounded and healing was evaluated by light microscopy (**B**) or were grown in soft agar as described in material and methods and three weeks later colonies formed were counted (**C**). The data represents the average ± SD of three independent experiments. *p < 0.05, **p < 0.01 vs pFNeo.

To further validate that CD43 promotes cell transformation leading to tumor development, and considering that the signaling capacity of CD43 depends on its intracellular domain [19], we transfected a mutant form of CD43 lacking the intracellular domain in A549 cells expressing endogenous CD43 and tested whether this mutant would work as a dominant negative molecule, reducing wound healing and anchorage-independent cell growth capacities. Noteworthy, for every fifty G418-resistant colonies obtained from transfecting the pFNeo empty vector, we obtained only one G418-resistant colony from transfecting the expression vector encoding the CD43 truncated mutant. Out of the four clones isolated, only two expressed the CD43 truncated mutant, suggesting that CD43 signaling is required for cell survival and proliferation of A549 tumor cells colonies. Accordingly, expressing the CD43 dominant negative mutant (Figure 3A) reduced both the wound healing (Figure 3B) as well as the anchorage-independent growth capacity of the lung derived A549 tumor cells (Figure 3C). These results validate our RNAi data and support the idea that CD43 signaling is required to promote cell transformation.

### CD43 overrides cell-cell contact inhibition of proliferation

**Figure 4:**
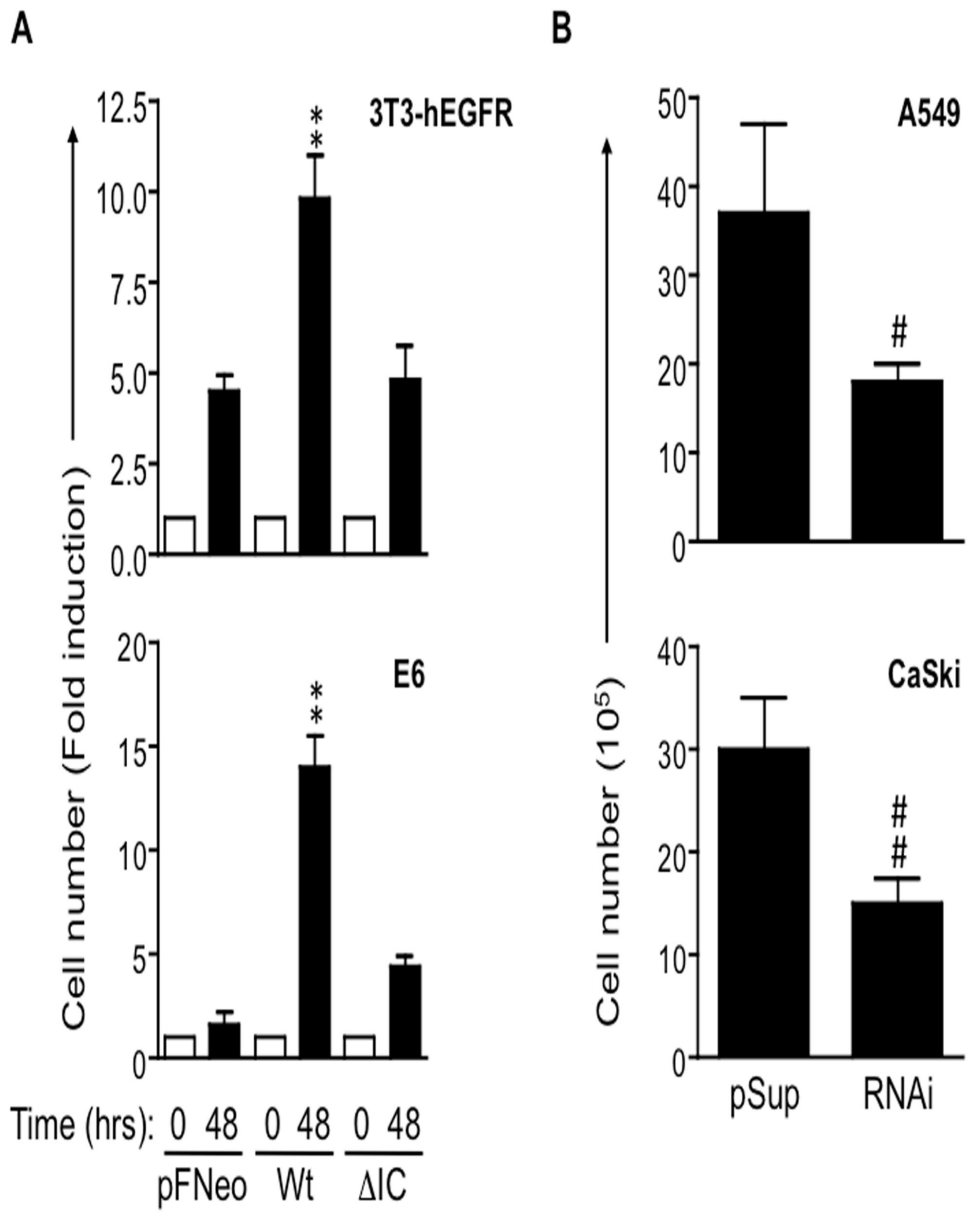
CD43 overrides cell-cell contact inhibition of growth. **A**) 2.5X10^5^ NIH-3T3-EGFR (3T3-hEGFR) or E6/E7 transgenic mouse fibroblasts (E6) carrying the pFNeo empty vector (pFNeo), expressing wild-type CD43 (Wt) or CD43 lacking the intracellular domain (ΔIC) were seeded, grown to confluence (t=0) and counted; duplicated plates were further cultured for 48 hrs and counted. **B**) 3X10^5^ A549 lung tumor cells or CasKi cervix tumor cells containing the empty pSuper (pSup) vector or expressing the CD43 specific RNAi (RNAi) were cultured to confluence and counted 48 hrs later. Data shown represent the average cell number ± SD of three independent experiments, including at least three independent clones of each cell line. *p < 0.05, **p < 0.01 vs pFNeo #p < 0.05 vs pSup).

Cell transformation is a complex process that involves a number of genetic changes that alter key biological functions [1] among which, cell proliferation. To test whether CD43 promoted cell proliferation, A549 or CasKi clones carrying an empty vector or a vector expressing a CD43-specific RNAi were seeded at subconfluent concentrations in supplemented medium and counted every day for four days. Under those conditions, we found no significant differences in cell numbers between cells with normal or reduced CD43 expression levels (Figure S5). This result was consistent with the fact that CD43 expression in NIH-3T3-hEGFR fibroblasts did not enhance EGFR or ERK activation following EGF exposure (Figure S6), suggesting that under optimal growth conditions, with sufficient mitogenic signals and space for cellular growth, the CD43-dependent signals are dispensable for cell proliferation. However, considering that tumor cell proliferation occurs at low growth factors concentrations and despite contact inhibition mechanisms of cell proliferation [1], we tested whether under adverse conditions, CD43 proliferative signals could be uncovered. NIH-3T3-hEGFR fibroblasts or E6/E7 transgenic fibroblasts expressing equivalent amounts of wild type CD43 or CD43 lacking the intracellular domain were allowed to reach confluence and further cultivated for an additional 48 hrs, and cell numbers were determined. Under these growth conditions, expression of wild-type CD43 favored cell proliferation of fibroblasts expressing the EGFR (Figure 4A, upper panel) as well as the HPV16 E6/E7 oncoproteins (Figure 4A, lower panel). As expected, the intracellular domain was required for the cells to proliferate under confluence since cells expressing the CD43 mutant lacking the intracellular domain proliferated at the same rate as cells carrying the empty vector (Figure 4A, upper and lower panels). Consistent with this, and in contrast to overexpressing CD43, reducing CD43 expression levels in A549 or CasKi tumor cells impaired cell proliferation under confluence (Figure 4B), independent of the tumor cell origin. All together, these results suggest that CD43 signals promote cell proliferation in a cell confluence-dependent manner. 

### CD43 signaling targets the Merlin pathway

Data shown above led us to hypothesize that the CD43-dependent signaling targets cell-cell contact inhibition of proliferation mechanisms. Recent experimental data suggest that under confluent conditions the intracellular domain of the CD43 molecule is cleaved, apparently by a γ-secretase dependent process [26], and translocates to the nucleus where it interacts with β-catenin/LEF complexes, promoting Cyclin D and Myc expression [13]. To test whether Cyclin D or c-Myc were involved in the CD43-induced cell proliferation under confluent conditions, we evaluated the Cyclin D and c-Myc protein levels at different time points after confluence of A549 tumor cells expressing normal or reduced CD43 levels. Cyclin D and c-Myc levels decreased in a time-dependent manner after confluence in A549 cells independently of the CD43 expression levels (Figure S7). However, unlike c-Myc levels, which were similar between cells with normal or reduced CD43 expression, cells with reduced CD43 expression had lower Cyclin D levels than cells with normal CD43 expression, suggesting that c-Myc does not play a major role in the CD43-promoted proliferation of confluent A549 cells.

**Figure 5:**
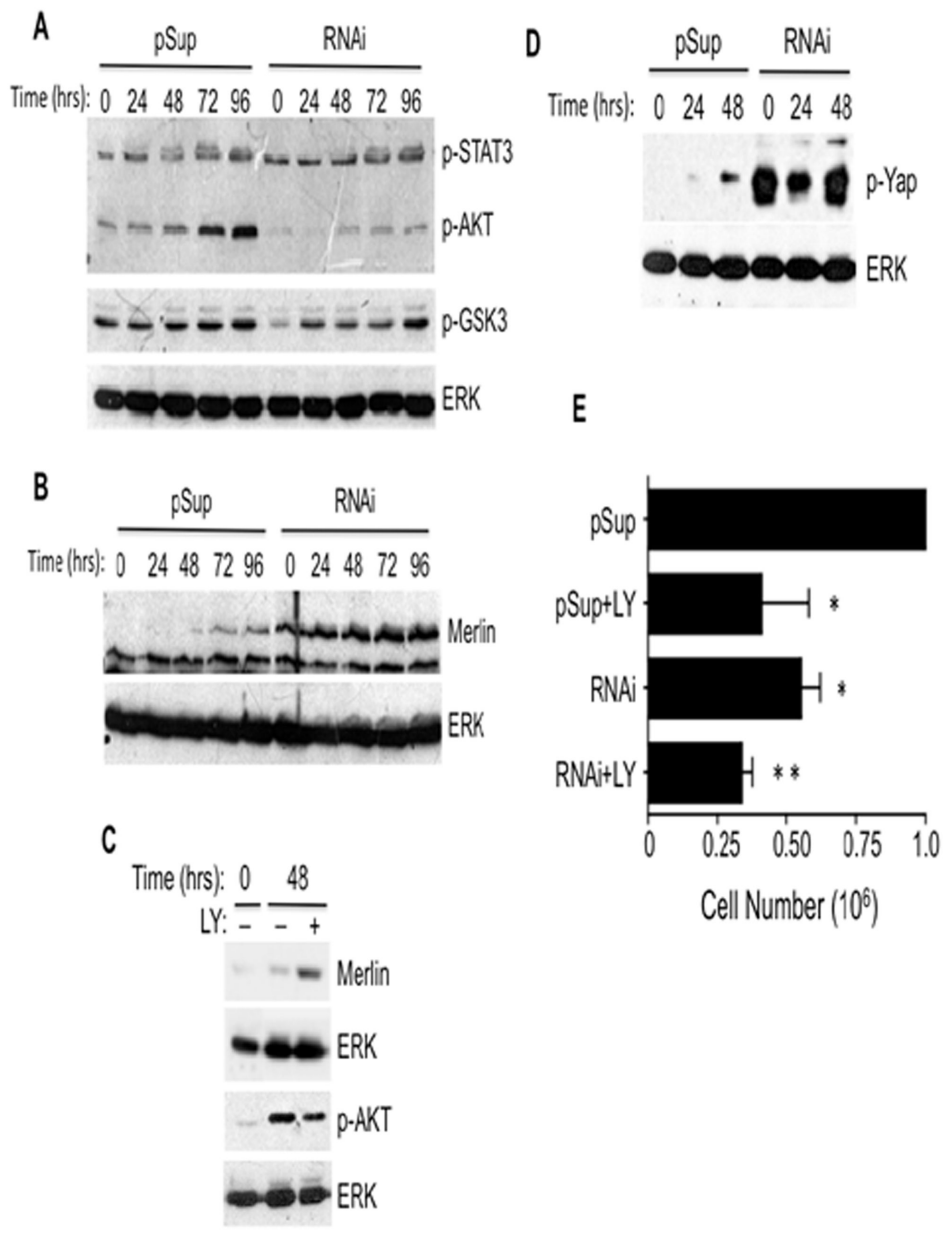
CD43 signaling targets the Merlin pathway. A549 clones containing the empty pSuper (pSup) vector or expressing the CD43 specific RNAi (RNAi) were grown to confluence (t=0) and further cultured for the indicated time points. At each time total cell extracts were prepared and the phosphorylation levels of STAT3 (p-STAT3), AKT (p-AKT), and GSK3β (p-GSK3) (**A**) as well as total Merlin levels (**B**) were determined by immunoblot, using specific antibodies. ERK protein levels were determined as loading control. **C**) Total cell extracts from A549 lung tumor cells cultured to confluence (t=0) or further cultured for 48 hrs in the absence (-) or presence of 20 μM LY294002 were resolved by SDS-PAGE and Merlin protein levels (Merlin) or phosphorylated AKT (pAKT) were evaluated by immunoblot with specific antibodies. ERK protein levels were used as loading control. **D**) A549 clones containing the empty pSuper (pSup) vector or expressing the CD43 specific RNAi (RNAi) were grown to confluence (t=0) and cultures were maintained for the indicated time points. Total cell extracts were prepared and the phosphorylation levels of YAP (p-YAP), ERK protein levels (loading control) were determined by immunoblot using specific antibodies. **E**) A549 clones expressing the empty pSuper (pSup) vector or the CD43 specific RNAi (RNAi) were grown to confluence and cultures were maintained in the absence or presence of 20 μM LY294002 (+LY) for 48 hrs. Cells were then harvested and counted. The graph represents the average cell number ± SD of three independent experiments using at least three independent clones. *p < 0.05, **p < 0.01 vs pSup.

The PI3K/AKT pathway positively regulates cell proliferation by regulating cell cycle inhibitors like p21 [27] and indirectly, by favoring the β-catenin pathway [28]. Data from our laboratory indicate that CD43 engagement activates the PI3K/AKT pathway in human T cells ([22], Bravo, ME and Sandoval, M, unpublished data). To evaluate whether CD43 signals activate this pathway restraining cell-cell contact inhibition of proliferation, we assessed AKT activation in A549 cells expressing normal or reduced CD43 levels at different time points after cells reached confluence. Interestingly, active phosphorylated AKT levels increased in confluent A549 cells expressing normal levels of CD43 but not in cells with reduced CD43 expression (Figure 5A). Accordingly, cells with low CD43 expression had less phosphorylated GSK3β, compared to cells expressing normal CD43 levels (Figure 5A, middle panel). These results indicate that in A549 lung tumor cells, CD43 promotes AKT activation. 

To identify the AKT targets that participate in the CD43-dependent effect on proliferation under cell confluence conditions, we first evaluated STAT3 phosphorylation, as this molecule has been suggested to override the cell-cell contact inhibition of proliferation [29]. STAT3 phosphorylation increased with time after cell reached confluence, however there was no difference in the phosphorylation kinetics between cells expressing normal or reduced CD43 levels (Figure 5A). As recently shown, Merlin is also an AKT substrate and Merlin AKT-mediated phosphorylation results in its degradation [30]. To determine whether CD43 signals lead to Merlin degradation we evaluated Merlin protein levels in A549 cells with normal or reduced CD43 expression at different time points after cells reached confluence. In contrast with A549 cells expressing normal CD43 levels, Merlin levels were higher in confluent cells with reduced CD43 expression, and increased over time (Figure 5B). 

To determine whether the PI3K/AKT pathway mediated the reduction in Merlin protein levels detected in A549 cells expressing normal CD43 amounts, confluent A549 cells were treated with the PI3K inhibitor LY294002 for 48 hrs and Merlin levels were evaluated by immunoblot. Inhibition of the PI3K/AKT pathway led to increased Merlin protein levels in confluent A549 cells (Figure 5C). Furthermore, in order to establish whether CD43 participated in the AKT-mediated phosphorylation of Merlin, we compared the extent of phosphorylated Merlin in A549 cells expressing normal or reduced CD43 levels. Consistent with data shown above, the p-Merlin levels observed in Merlin immunoprecipitates from A549 with normal CD43 expression were higher than those observed in immunoprecipitates from LY294002-treated cells or those of cells with reduced CD43 expression (Figure S8). Together, these results indicate that the CD43-dependent AKT activation leads to Merlin phosphorylation and degradation. 

Since Merlin regulates cell proliferation by inhibiting YAP-dependent transcription through YAP phosphorylation [7], we evaluated YAP phosphorylation levels in total cells extracts prepared from confluent A549 cells expressing normal or reduced CD43 levels. In agreement with the elevated Merlin levels found in confluent A549 cells with reduced CD43 expression, YAP phosphorylation levels were clearly higher in those cells as compared with confluent A549 cells expressing normal CD43 levels (Figure 5D). Moreover, inhibition of the PI3K/AKT pathway impaired the capacity of A549 cells to proliferate under confluence (pSup+LY) to levels comparable to those of A549 cells with reduced CD43 expression levels (RNAi) (Figure 5E). Reduction in CD43 expression and inhibition of the PI3K/AKT pathway (RNAi+LY) did not result in further blockade of cell proliferation than reducing CD43 expression (RNAi) or inhibiting the PI3K/AKT pathway (pSup+LY) independently (Figure 5E). The activation of this pathway was dependent of cell density since in low cell density cultures we did not detect differences in total Merlin protein levels nor in phosphorylated AKT or phosphorylated YAP levels between cells with reduced CD43 expression (RNAi) and cells with normal CD43 expression (Figure S9) 

**Figure 6:**
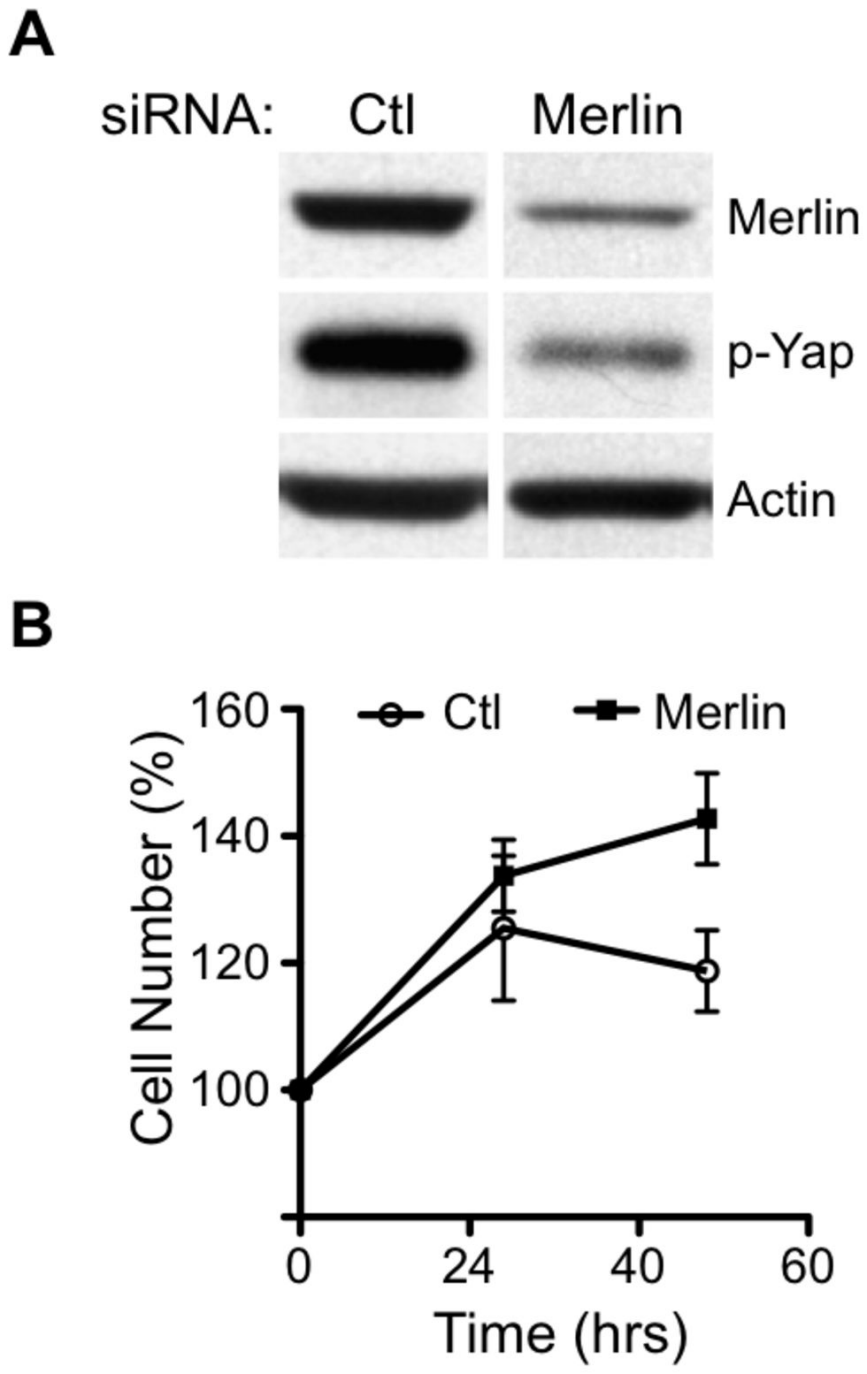
Merlin mediates the inhibition of A549 cell proliferation by cell-cell contact. **A**) A549 clones expressing the CD43 specific RNAi (RNAi) were transfected with non-specific (Ctl) or Merlin specific siRNAs (Merlin). 48 hrs after cells reached confluence total cell extract were prepared and Merlin protein levels (Merlin) as well as phosphorylated Yap levels (p-YAP) were evaluated by immunobloting using specific antibodies. Actin levels were used as loading control. **B**) The proliferation capacity of A549 clones expressing the CD43 specific RNAi (RNAi) transfected with non-specific (Ctl) or Merlin specific siRNA (Merlin) was evaluated at the indicated time points after cells reached confluence. Data represent the average of three independent experiments using two different clones.

To further confirm that Merlin restricts cell proliferation when CD43 expression is compromised, we reduced Merlin protein levels by siRNAs in clones of the A549 cell line with low CD43 expression levels and evaluated their proliferative capacity after confluence. Transfection of the specific siRNAs for Merlin resulted in a clear reduction in Merlin protein levels compared with cells transfected with a nonspecific siRNA (Figure 6A, upper panel), accordingly the levels of phoshorylated YAP were also reduced as compared to cells expressing normal Merlin protein levels (Figure 6A, middle panel). In agreement with the reduction in Merlin expression and YAP phosphorylation, cells transfected with the Merlin specific siRNA showed a enhanced proliferation capacity under confluence conditions compared with control siRNA transfected cells (Figure 6B). Altogether, these results suggest that CD43 signaling overrides cell-cell contact inhibition of proliferation via AKT-mediated Merlin phosphorylation and degradation. 

## Discussion

Cell transformation and tumor development is a multistep process that involves alterations in key cell functions, including cell proliferation and survival. As a result from increased proliferative signaling, defective inhibitory mechanisms, or both, most tumors show enhanced cell proliferation. The Hippo pathway controls proper cell number and tissue size in the adult organism, a mechanism known as cell-cell contact inhibition of proliferation and the product of the Nf2 gene, Merlin, is a key player in this process. Not surprisingly, many tumors carry deleterious mutations in this gene [31,32], leading to constitutive activation of the Merlin target, the YAP transcriptional coactivator [33]. However, consistent with the fact that not all tumors carry a mutated Nf2 gene, distinct signaling pathways that inactivate the Hippo pathway and favor tumor development have been described [4,30,34,35].

Independent studies have documented CD43 expression in different non-lymphoid human tumors derived from lung, cervix, breast, and colon [10,24,36–38], yet the role of this molecule in cell transformation and carcinogenesis remains largely unknown. Here, we showed that CD43-induced signals cooperate with distinct oncogenic signals to boost the transformation potential of murine fibroblasts. Likewise, we found that CD43 expression in human lung-, cervix- and colon-derived cancer cells enhances cell motility, proliferation, anchorage-independent growth and tumor formation. At the molecular level, we showed that CD43 signaling targets the Hippo pathway by promoting Merlin degradation, overriding cell-cell contact inhibition of proliferation and promoting carcinogenesis.

**Figure 7:**
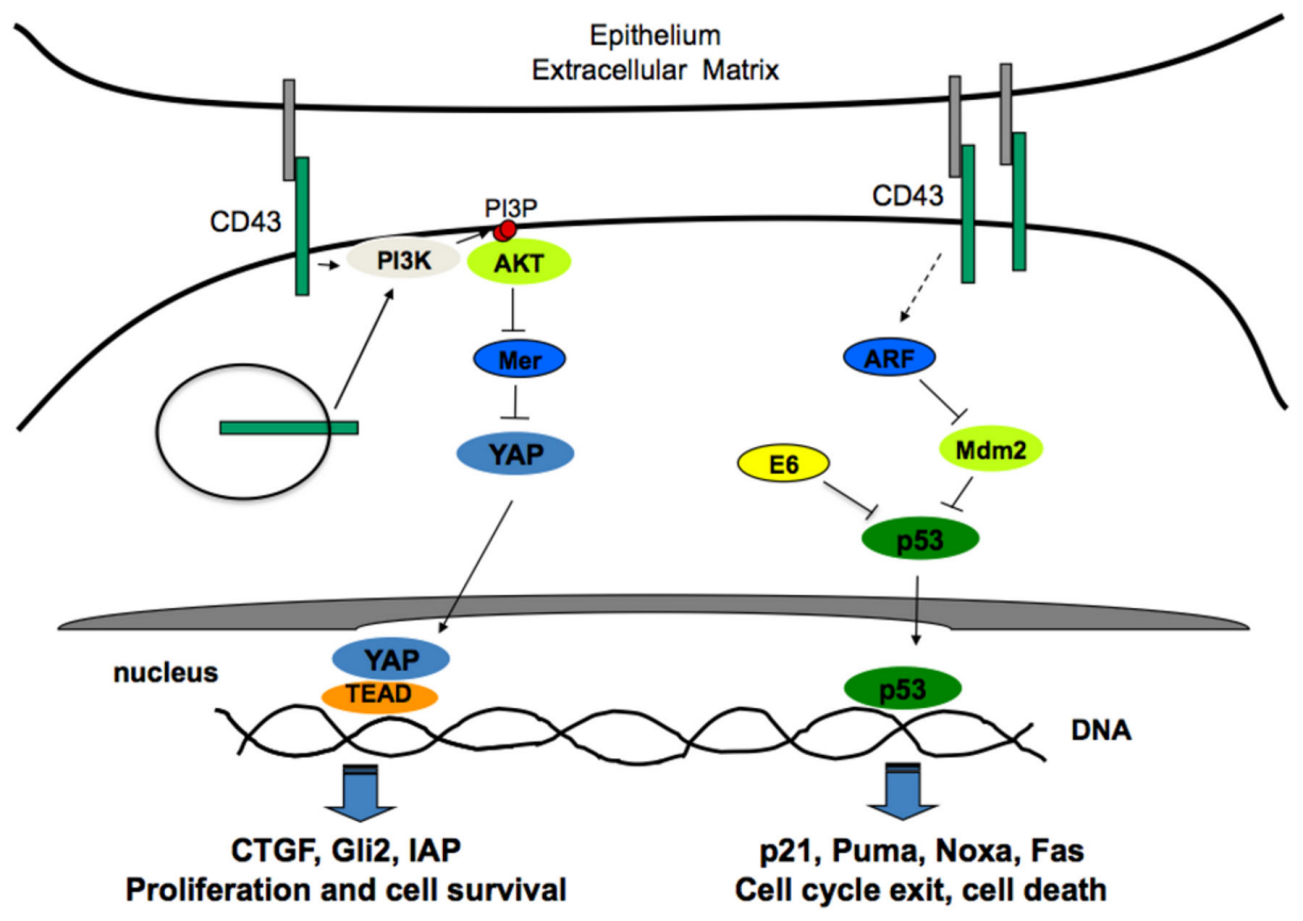
CD43 signaling cooperates with oncogenic signals to promote cell transformation. In epithelial cells, upon interaction with putative ligand(s) present on the cell surface of neighboring cells or in the extracellular matrix, CD43 activates the PI3K/AKT pathway that results in the inhibition of the Hippo pathway by a mechanism involving Merlin phosphorylation and degradation, thus favoring cell survival and proliferation. Depending of the cellular context, signaling from intracellular CD43 located either on membrane vesicles or the nuclear membrane may also contribute to cellular transformation. Nonetheless, this might not be sufficient to overcome the anti-proliferative and death effects resulting from the activation of the ARF-p53 pathway also induced by CD43 [24]. However, in transformed cells with an impaired p53 pathway resulting from oncogenic signals like those provided by the E6 oncoprotein from HPV16, CD43 signaling promotes cells proliferation and tumor formation.

Previously published data showed that CD43 expression in cells carrying a defective ARF/p53 pathway results in cell proliferation [23], while, in cells expressing wild-type p53, CD43 expression results in apoptotic cell death [24], suggesting that CD43 expression is not sufficient to promote cell proliferation and that other oncogenic alterations are necessary. In line with this idea, herein we showed that expression of CD43, in combination with oncogenic signals resulting from EGFR over-expression or from the expression of the HPV16 E6/E7 oncoproteins, promoted motility, anchorage-independent growth and *in vivo* tumor formation of murine fibroblasts. This cooperative effect may result from the inactivation of p53 [39–41], and from preventing CD43-triggered apoptosis, two events imposed on by EGFR or E6/E7 signaling. This in turn could favor the establishment of a positive feedback loop whereby CD43 may contribute to amplify the transforming capacities of the EGFR or E6/E7 oncogenes, ultimately promoting cell transformation (Figure 7). 

Unlike G protein-coupled receptors that promote cell transformation by indirectly regulating EGFR activation through the release of EGF from the extracellular matrix [42], CD43 expression did not increase EGFR phosphorylation levels, suggesting that CD43 signals cooperate with the EGFR further downstream in the signaling cascade controlling cell growth. Despite the fact that EGF-induced cell growth involves ERK activation, and that CD43 has been shown to activate ERK in human peripheral T cells [43], CD43 signaling did not augment the EGFR-induced ERK activation, suggesting that the effect of CD43 on mouse fibroblasts cell proliferation is ERK-independent.

To confirm that CD43 expression confers a transforming advantage to non-lymphoid human derived cancer cells, we reduced endogenous CD43 expression levels by RNAi interference. We found that reducing CD43 expression in human lung-, cervix-, and colon-derived tumor cells impaired their motility, anchorage-independent growth as well as *in vivo* tumor formation. Although reducing CD43 expression did not prevent tumor formation, a clear reduction in tumor mass was observed, independently of the tumor cell origin. Moreover, the fact that expressing a dominant negative form of the CD43 molecule in the lung-derived tumor cell line A549 prevented cell motility and anchorage-independent growth, and that only a few colonies expressing the CD43 mutant molecule were recovered as opposed to when the empty vector was transfected (4 versus150), points to a role for CD43 signals in the proliferation and survival of lung-derived tumor A549 cells. This is in agreement with recent results indicating that CD43 expression in A549 cell prevents TNF-α-induced apoptosis and NK cells-mediated cell lysis [44].

In human colon-derived tumor cells, CD43 signaling leading to cell proliferation has been reported to involve the proteolytic cleavage of the CD43 intracellular domain, its nuclear translocation and interaction with the TCF/LEF1/β-catenin molecular complex, thus resulting in c-Myc and Cyclin D expression [13]. In contrast, we found that c-Myc levels were comparable in A549 lung cells with normal or reduced CD43 expression when grown in confluence. Since CD43 expression was not entirely reduced in the clones carrying the CD43 specific RNAi, it is possible that there is still enough CD43 to be cleaved to support c-Myc expression. However, the fact that reducing CD43 expression severely impaired the activation of the AKT pathway, which regulates the β-catenin-mediated c-Myc expression [28], suggest that c-Myc is not involved in the CD43-dependent proliferation of confluent A549 cells. The fact that Cyclin D protein levels were reduced in cells with low CD43 expression compared with that observed in cells with normal CD43 expression, supports that CD43 signaling promotes Cyclin D expression in lung-derived tumor cells, independently of the β-catenin pathway. Consistent with this, Merlin has also been shown to negatively regulate cyclin D expression [45]. A possible explanation for the discrepancy between our results and those from Andersson et al. [13] and Baikova et al [46] may be explained by the cellular context as colon epithelium transformation depends largely on the activation of the β-catenin pathway [47], while lung tumor development has not been strongly associated to activation of this pathway. However, nuclear localization of the full length CD43 molecule seem to play an important role in cells transformation independently of the tumor cell origin since, full length CD43 has been observed in the nuclei of colon and lung tumor cell lines [44,46]. Independently of the molecular mechanism underlying nuclear CD43-dependent cells transformation, our results obtained with the lung CD43^+^ A549 cell line over-expressing the CD43 mutant lacking the intracytoplamic domain further support the idea CD43 signaling through its intracytoplamic domain is key to promote cell transformation (Figure 7). 

Although, STAT3 activation resulting from increased EGFR signaling has been implicated in the development of lung adenocarcinomas [29], the cell-cell contact-induced STAT3 phosphorylation was not affected by CD43 signaling since STAT3 phosphorylation levels were comparable in A549 cells with impaired CD43 expression as in wild-type cells. However, we found that instead of depending upon STAT3 to bypass cell-cell contact inhibition of proliferation, CD43 expression overrides cell-cell contact inhibition of proliferation by promoting Merlin degradation. Impairment of CD43 expression by RNAi in A549 cells severely reduced the cell-cell contact-dependent activation of the PI3K/AKT pathway, resulting in increased Merlin protein levels and cell growth inhibition. This effect was Merlin-dependent since knocking down Merlin protein levels restored the proliferative capacity of A549 cells with reduced CD43 expression, under space-limiting conditions. This involved PI3K activity since the LY294002 inhibitor enhanced Merlin protein levels in confluent A549 cells expressing endogenous CD43. Consistent with data showing that Merlin phosphorylation by AKT tags it for ubiquitylation and degradation [30], Merlin was phosphorylated in A549 cells expressing normal CD43 levels in a PI3K/AKT-dependent manner since the LY294002 inhibitor reduced the Merlin phosphorylation levels as compared to cells with reduced CD43 expression. Inhibition of the PI3K/AKT pathway in A549 cells also increased YAP phosphorylation levels. YAP phosphorylation impairs its transcriptional activity and as a consequence, cell proliferation and survival [7]. Accordingly, cell-cell contact inhibition of proliferation was restored when A549 cells expressed reduced levels of CD43 and consequently exhibited reduced AKT activity or when the PI3K/AKT pathway was blocked with the LY294002 inhibitor in A549 cells with normal CD43 expression. Collectively, these data further supports the hypothesis that CD43 signaling leads to PI3K/AKT activation, promoting Merlin phosphorylation and degradation, which results in activation of the YAP transcriptional regulator, ultimately leading to sustained expression of genes involved in cell proliferation and survival. However our results do not preclude the possibility that in non-epithelial cells, CD43 promotes cells proliferation in a confluence-dependent manner by cooperating with the β-catenin pathway rather than regulating Merlin. Thus the mechanism by which CD43 promotes cell proliferation under space limiting conditions may be cell context-dependent.

Interestingly, Merlin is also involved in preventing cell motility [48], and the PI3K/AKT pathway may also negatively regulate this cellular function of Merlin [35]. The fact that expression of the CD43 dominant negative mutant, reduction of CD43 expression or inhibition of the PI3K/AKT pathway all impaired cell migration, suggests that CD43 signaling is also modulating the Merlin-mediated regulation of cell migration. In line with these data, previously published results indicate that CD43 interaction with E-selectin favors tumor metastasis [14] and cell adhesion to human microvascular endotelial cells and cell migration [44]. It is conceivable that at early stages of the transformation process, CD43-mediated signals contribute to impair the Hippo pathway, resulting in clear proliferative and survival advantages and that at later stages, CD43 signaling promotes cell metastasis by altering cell-cell interactions and cell migration. Therefore, as in the case of lymphoid tumors [49], CD43 expression in non-lymphoid tumor should also be considered as a factor for poor prognosis. 

## Supporting Information

Figure S1 **CD43 expression promotes anchorage independent cell growth.**
(TIFF)

Figure S2 **EGFR and CD43 expression in different fibroblast clones.**
(TIFF)

Figure S3 **In the absence of oncogenic signals CD43 expression does not promote cell transformation.**
(TIFF)

Figure S4 **Down modulation of CD43 expression impairs tumor growth.**
(TIFF)

Figure S5 **CD43 expression does not affect tumor cell proliferation.**
(TIFF)

Figure S6 **CD43 expression does not alter EGFR signaling.**
(TIFF)

Figure S7 **CD43 expression does not affect c-Myc or cyclin D protein levels in A549 confluent cells.**
(TIFF)

Figure S8 **CD43 expression promotes Merlin phosphorylation.**
(TIFF)

Figure S9 **Under low cell density CD43 signaling does not affect the AKT-Merlin pathway.**
(TIFF)
