# Supplementary figures and images for: CD43 Promotes Cells Transformation by Preventing Merlin-Mediated Contact Inhibition of Growth
Source: PLoS One. 2013 Nov 18;8(11):e80806. doi: 10.1371/journal.pone.0080806 (PMC3832598; doi:10.1371/journal.pone.0080806)

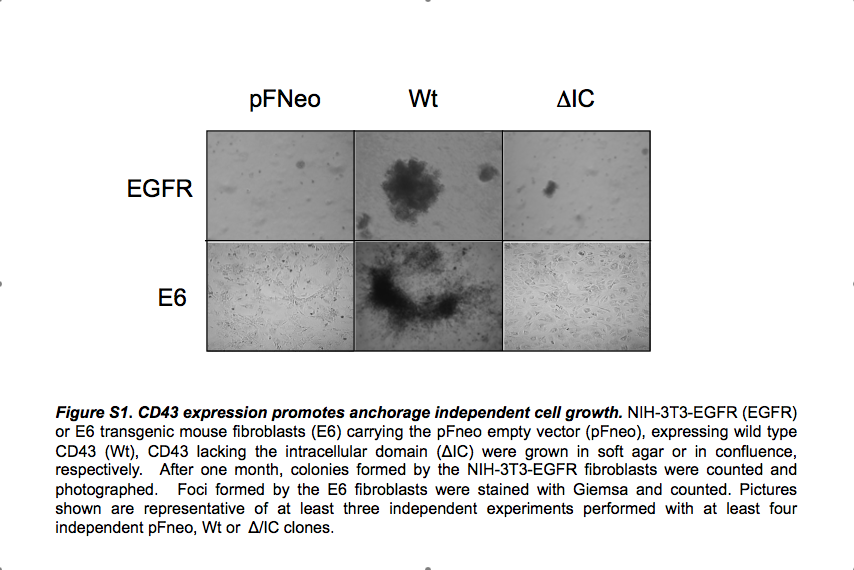

Supplement: Figure S1 — CD43 expression promotes anchorage independent cell growth. (TIFF) [file pone.0080806.s001.tiff]

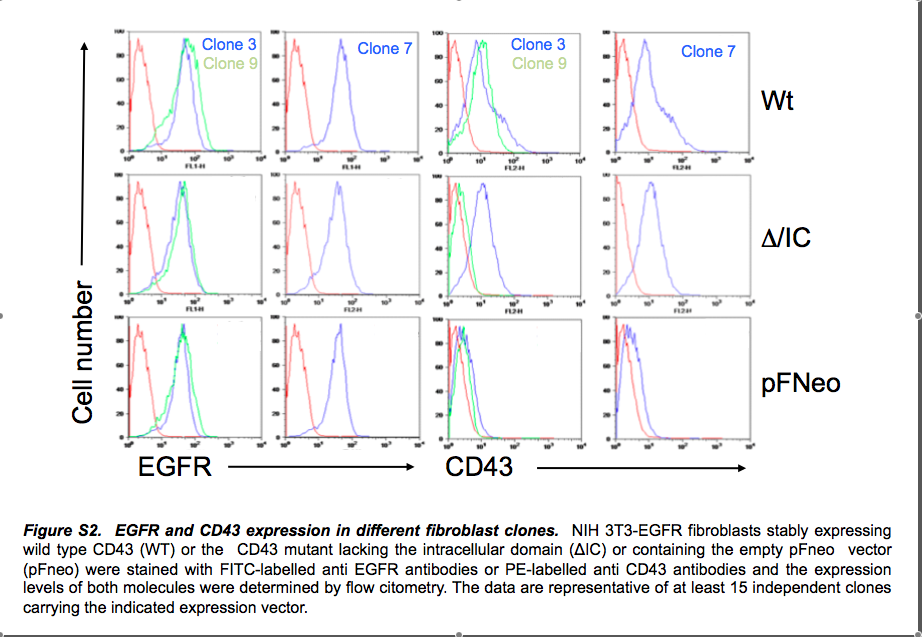

Supplement: Figure S2 — EGFR and CD43 expression in different fibroblast clones. (TIFF) [file pone.0080806.s002.tiff]

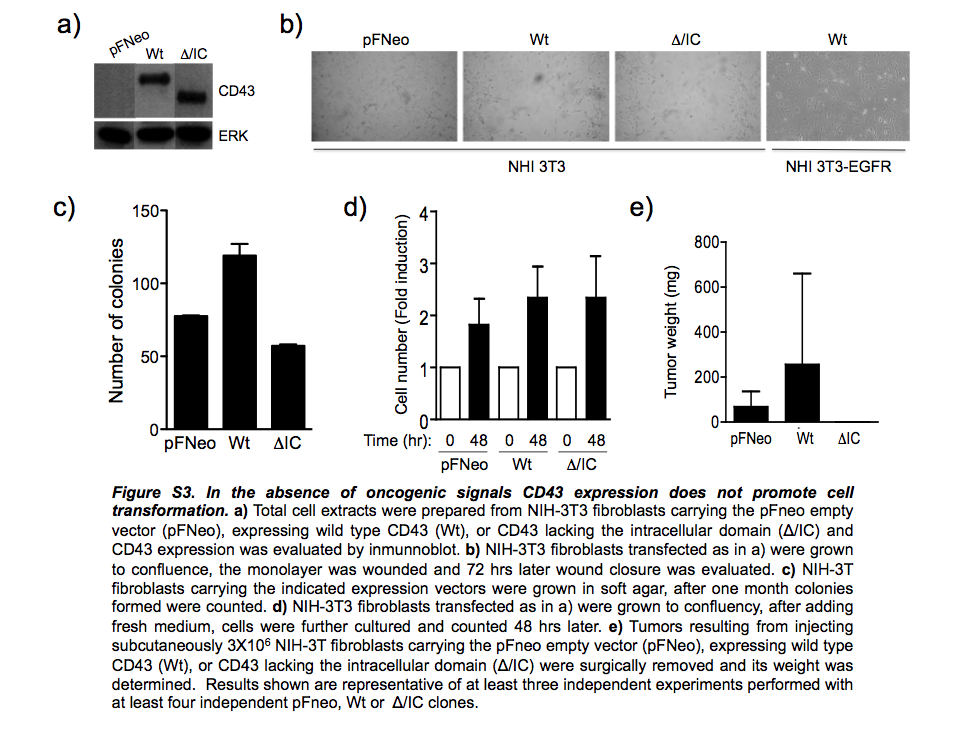

Supplement: Figure S3 — In the absence of oncogenic signals CD43 expression does not promote cell transformation. (TIFF) [file pone.0080806.s003.tiff]

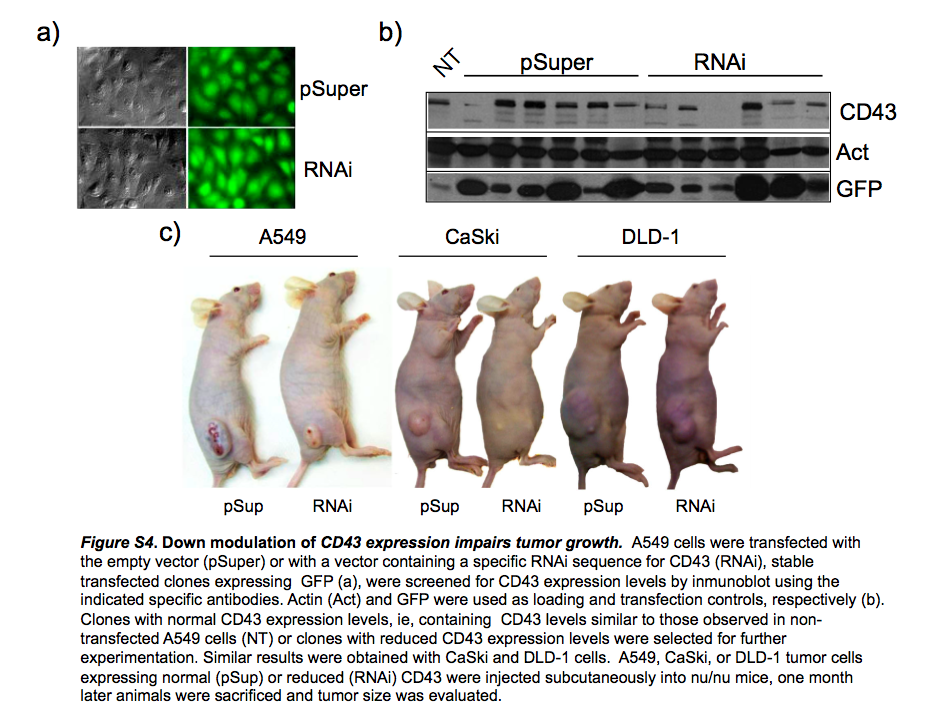

Supplement: Figure S4 — Down modulation of CD43 expression impairs tumor growth. (TIFF) [file pone.0080806.s004.tiff]

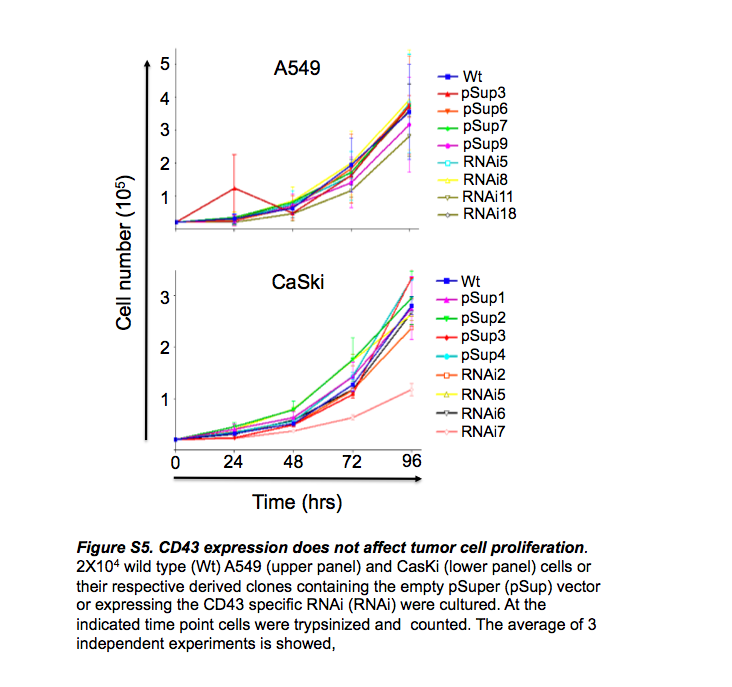

Supplement: Figure S5 — CD43 expression does not affect tumor cell proliferation. (TIFF) [file pone.0080806.s005.tiff]

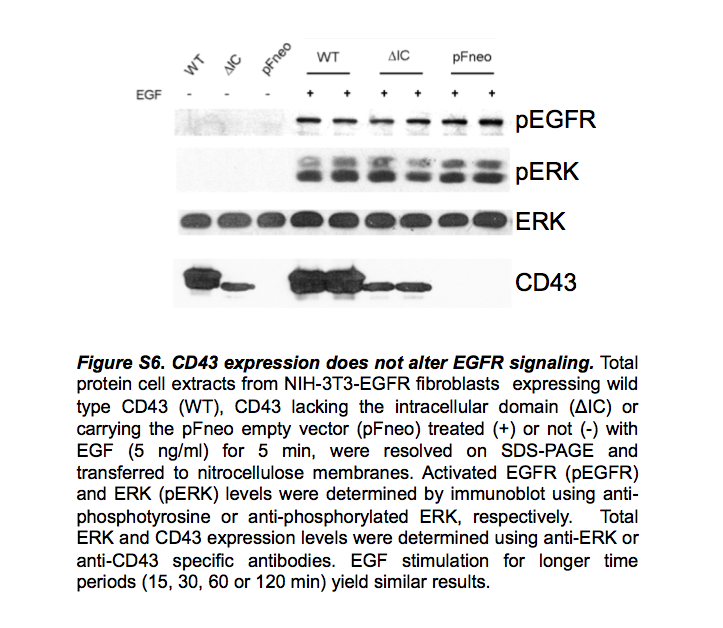

Supplement: Figure S6 — CD43 expression does not alter EGFR signaling. (TIFF) [file pone.0080806.s006.tiff]

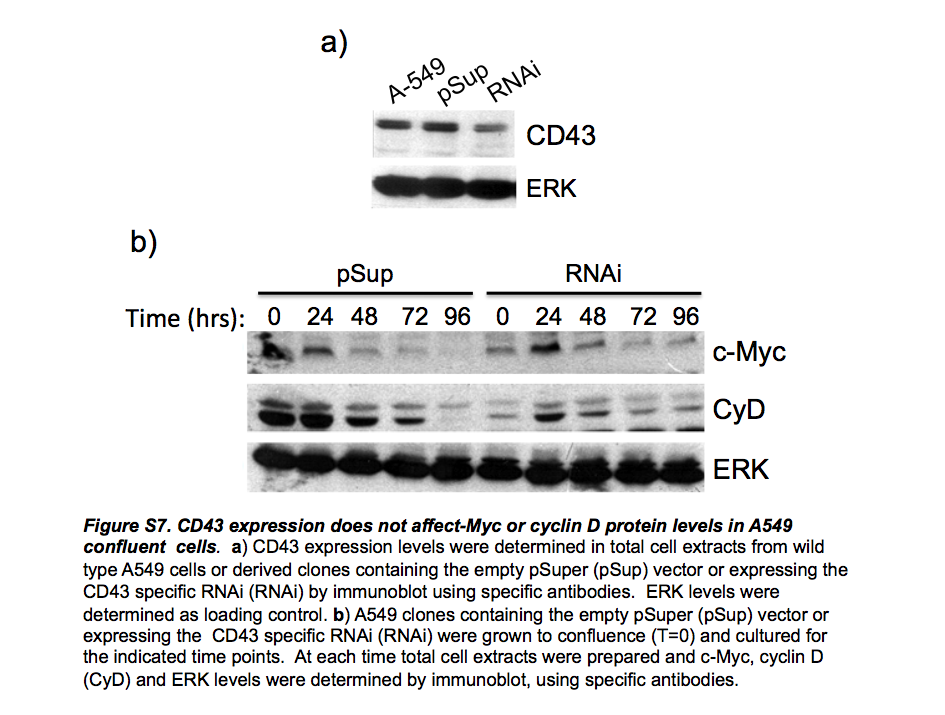

Supplement: Figure S7 — CD43 expression does not affect c-Myc or cyclin D protein levels in A549 confluent cells. (TIFF) [file pone.0080806.s007.tiff]

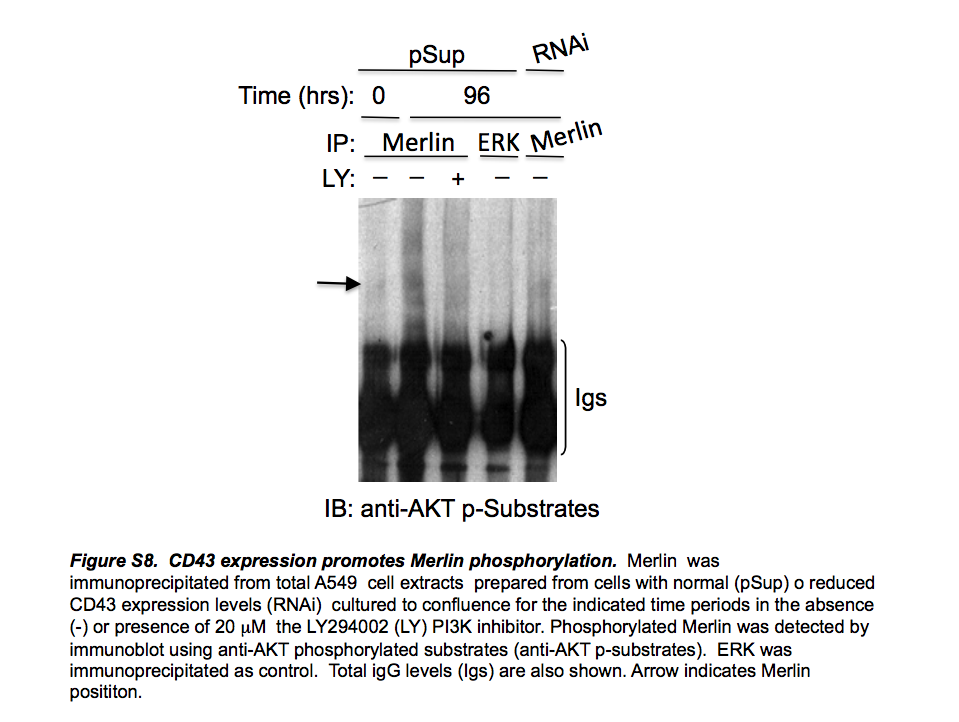

Supplement: Figure S8 — CD43 expression promotes Merlin phosphorylation. (TIFF) [file pone.0080806.s008.tiff]

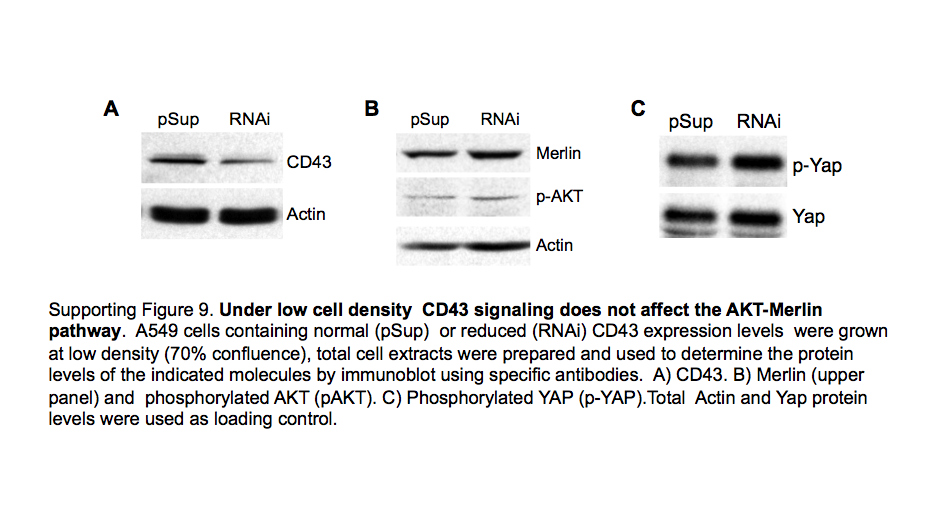

Supplement: Figure S9 — Under low cell density CD43 signaling does not affect the AKT-Merlin pathway. (TIFF) [file pone.0080806.s009.tiff]
